# Assessing the frontline competency gap: Emergency care perceptions among nurses in Yemen's conflict zone

**DOI:** 10.1111/inr.13047

**Published:** 2024-10-03

**Authors:** Zakaria Ahmed Mani, Adnan Innab, Fuad Taleb

**Affiliations:** ^1^ Nursing Department Jazan University Jazan Saudi Arabia; ^2^ Nursing and Midwifery Department Monash University Frankston Australia; ^3^ Nursing Administration, and Education Department, Vice‐Dean of Student Affairs, College of Nursing King Saud University Riyadh Saudi Arabia; ^4^ Department of Nursing, Faculty of Medicine and Health Sciences Taiz University Taiz Yemen; ^5^ Nursing Department Vision Colleges Riyadh Saudi Arabia

**Keywords:** Accident and emergency nursing, disaster nursing, disaster nursing education, education, nursing, nursing assistant education

## Abstract

**Background:**

Nursing competencies in armed conflict situations are critical for effective response and recovery. This study explores nurses’ perceptions regarding their competencies in armed conflict zones to identify areas of proficiency and those requiring further emphasis in training.

**Methods:**

This cross‐sectional descriptive study adhered to the STROBE reporting guidelines. It used a validated questionnaire examining 47 different competencies for nursing in armed conflict zones, which were subsequently ranked to identify those that were valued most and least.

**Results:**

In total, 102 questionnaires were returned (85% response rate). The highest‐ranked competencies focused on immediate life‐saving interventions and personal safety. In contrast, competencies involving broader disaster management, such as understanding organizational disaster plans, post‐death care, and risk identification, ranked lower. This observation may indicate a tendency to prioritize direct clinical care over strategic planning and long‐term recovery in disaster nursing education.

**Conclusion and implications for nursing and health policy:**

This study highlights the critical need to strengthen emergency care competencies among nurses working in armed conflict zones in Yemen. Its findings underscore the importance of targeted training programs, particularly in complex trauma management and psychological first aid, to address nurses’ self‐identified competency gaps. Policy implications include prioritizing resource allocation for emergency care infrastructure, implementing competency‐based deployment strategies, and ensuring access to mental health support for nurses in working conflict zones. These actions are essential for building a resilient nursing workforce capable of providing quality care amidst the unique challenges of armed conflict.

## INTRODUCTION

Human‐generated conflicts create significant health crises, marked by increased cases of illnesses, injuries, and deaths along with overwhelming numbers of afflicted people (Levy & Sidel, 2016; Mani et al., 2020, 2024; Trelles Centurion et al., 2017). Notably, conflicts, particularly in Syria and other nations such as Afghanistan, Iraq, Nigeria, and Ukraine, resulted in the largest number of deaths since World War II in 2014. The conflict in Yemen, which began in 2014, has severely impacted its healthcare systems (Uppsala Conflict Data Program, 2024). These conflicts have multiple adverse effects on emergency departments, where personnel are strained and facilities are overcrowded due to the influx of patients injured in the conflicts and other medical emergencies, leading to prolonged wait times (Bou‐Karroum et al., 2020; Levy & Sidel, 2016; Mani et al., 2023b).

Yemen has been grappling with protracted armed conflicts that have had a devastating impact on its healthcare systems and the well‐being of its population (AlKarim et al., 2021; Bou‐Karroum et al., 2020; Elnakib et al., 2021; Geneva Declaration Secretariat, 2015; Human Rights Watch, 2015). The ongoing conflicts have resulted in the widespread destruction of healthcare services, critical shortages of medical supplies and personnel, and severely limited access to essential healthcare services (Elnakib et al., 2021; Human Rights Watch, 2015; International Medical Corps, 2024; Levy & Sidel, 2016). This dire situation is further exacerbated by the breakdown of social structures, economic instability, and mass displacement, leaving communities extremely vulnerable to health risks (Geneva Declaration Secretariat, 2015; International Medical Corps, 2024; World Health Organization, 2024).

As the largest healthcare workforce globally, nurses are at the forefront of providing care in these challenging environments (Houser, 2016; International Council of Nurses, 2019; Veenema et al., 2016). They face immense pressure to deliver quality care amidst immense adversity, often working in resource‐constrained settings, facing security threats, and witnessing traumatic events (Elnakib et al., 2021; Jabbour et al., 2021; Loke & Fung, 2014). The psychological toll on nurses working in conflict zones is significant, with high rates of burnout, compassion fatigue, and post‐traumatic stress disorder reported (AlKarim et al., 2021; Elnakib et al., 2021; Turale, 2017). Therefore, understanding the specific challenges nurses face in conflict zones such as Yemen and ensuring they are adequately trained and supported is crucial for delivering effective healthcare services and building resilient health systems in these vulnerable regions (International Council of Nurses, 2019).

The growing global unrest has highlighted a gap in nursing education: the lack of disaster management training, particularly concerning armed conflicts (Mani et al., 2024; Said & Chiang, 2020; Usher et al., 2015). Modern warfare tactics have evolved to commonly include chemical, biological, radiological, nuclear, and explosive agents (Mani et al., 2023b; Veenema et al., 2016). However, nursing curricula have not adequately adapted to these changes (Bou‐Karroum et al., 2020; Murphy et al., 2021). Consequently, emergency nurses often find themselves inadequately prepared to respond to the unique challenges posed by armed conflicts (Bou‐Karroum et al., 2020; Elnakib et al., 2021; Loke & Fung, 2014; Mani et al., 2024). In addition to this burden, the healthcare system in Yemen has been severely disrupted by the ongoing conflicts, with facilities damaged or destroyed, medical supplies and equipment scarce, and healthcare workers displaced or unable to report to work due to security concerns (Geneva Declaration Secretariat, 2015; World Health Organization, 2024). These issues have significantly burdened the remaining healthcare workers, including nurses, who must provide care under extremely challenging conditions (Geneva Declaration Secretariat, 2015; World Health Organization, 2024).

To better understand the experiences and perspectives of nurses working in armed conflict zones, this study explores the perceptions of Yemeni nurses regarding their competencies in providing care during the ongoing armed conflict. It has significant implications for strengthening disaster nursing preparedness, particularly in conflict‐ridden regions such as Yemen and the Middle East, where healthcare systems are constantly challenged by armed conflict. This research can provide valuable insights for developing targeted training programs that cater to the specific needs of nurses with varying experience levels. Ultimately, its findings can inform policy decisions and resource allocation within healthcare systems to ensure that nurses are adequately prepared to deliver effective and timely care during armed conflicts and other disasters, ultimately contributing to improved health outcomes in these vulnerable populations.

## METHODS

### Design

This cross‐sectional descriptive study adhered to the Strengthening the Reporting of Observational Studies in Epidemiology (STROBE) guidelines.

### Setting and sampling design

The study data were collected using a convenience sampling method. The study settings were two hospitals in Taiz, Yemen, that treated casualties of armed conflicts in that area: the Republican General Teaching Hospital and the Atharwa Authority Hospital. These hospitals have experienced frequent presentations of casualties from armed conflicts at different times. The study population comprised all nurses who had treated casualties of the armed conflict. Participants were selected through convenience sampling.

### Inclusion criteria

Nurses who held a minimum of a two‐year Diploma of Nursing qualification and had cared for casualties of the armed conflict in the two selected hospitals were eligible.

### Instrument

This study adopted a validated questionnaire for assessing nurses’ competencies in armed conflict zones (Mani et al., 2023b) that had been developed through various validated processes considering well‐established resources for disaster nursing competencies (Al Thobaity et al., 2016; International Council of Nurses, 2019; Mani et al., 2020). Its internal reliability was excellent (Cronbach's alpha = 0.986), and permission to use it was obtained from the corresponding author.

The validity of the adopted questionnaire was established through a two‐step pilot testing process. Two researchers with relevant expertise initially examined the questionnaire, proposing minimal adjustments. Then, a second pilot was undertaken with a panel of 10 experts who were likely to understand the subject matter: five disaster/emergency nursing experts with clinical and academic backgrounds and five emergency nurses working in hospitals relevant to this study. These panel members evaluated the questionnaire's content and clarity and assessed whether its questions effectively measured the intended constructs (Grove & Gray, 2018; Houser, 2016). The positive feedback provided for the questionnaire indicated its relevance and comprehensibility.

The questionnaire comprised six demographics questions (i.e., gender, hospital, department, age, hospital type, qualification, and range of experience), 47 competency questions explicitly developed for armed conflict zones, and one question asking for any comments or suggestions (Mani et al., 2023b). The questionnaire was established on the premise of “What do nurses need to be able to do?”(International Council of Nurses, 2019). Each competency measures a critical task that nurses should be capable of performing in their roles, each signifying an aspect of practical ability and theoretical knowledge essential for their professional duties (International Council of Nurses, 2019).

The questionnaire was translated from English into Arabic by an expert official translator and then back‐translated into English to ensure the fidelity of the Arabic translation. No changes were made.

Finally, a hard copy of the questionnaire was distributed to nurses in the two hospitals through charge nurses in different departments. As this was the first study to test the translated version of this questionnaire, its internal consistency reliability was assessed. Its Cronbach's alpha was 0.949, indicating excellent reliability.

### Data collection

After obtaining ethical approval from Taiz University, relevant nurses were recruited. Data collection was conducted by a coauthor from each hospital. Potential participants were provided with an explanatory statement and invited to anonymously complete and return the questionnaires to a sealed box placed in the nurses’ rest area. The coauthor emptied the boxes weekly for one month.

### Ethical considerations

This study was conducted according to the ethical principles of the Declaration of Helsinki. It was approved by the Ethical Committee of the Faculty of Medicine, Taiz University, Yemen (Ref. No: 046/23; date: December 10, 2023). The participants were provided with information about the study's purpose, procedures, and their right to withdraw at any time without any consequences. Completion of the questionnaire was considered as implied consent to participate. Confidentiality and anonymity were maintained throughout this study by deidentifying data and using secure storage methods.

### Data analysis

The data were entered into a Microsoft Excel spreadsheet and double‐checked by the authors. Then, the data were converted for analysis using SPSS software (version 29). Nursing competencies were ranked from highest to lowest based on their mean scores. Differences in core competencies according to participants’ demographic characteristics were examined using independent sample *t* tests and one‐way analysis of variance (ANOVA).

## RESULTS

### Demographic characteristics

Of the 120 distributed questionnaires, 102 completed questionnaires were returned, representing a response rate of 85%. Table 1 presents the participants’ demographic characteristics. Their age ranged from 20 to 50 years, with most (83.3%) aged 20−30 years. Most participants were female (54.9%), held a diploma (70.6%), had worked in the nursing profession for < 5 years (58.8%), and worked in public hospitals (51.0%). Participants worked in either the emergency room (ER) or surgical department (27.5%).

**TABLE 1:** Sample characteristics of participants (*N* = 102).

Variable (range)	*n* (%)
Age	
20–30	85 (83.3%)
31–40	16 (15.7%)
41–50	1 (1%)
Gender	
Male	46 (45.1%)
Female	56 (54.9%)
Level of nursing education	
Diploma	72 (70.6%)
BSN	30 (29.4%)
Length of experience, years	
<5	60 (58.8%)
5 to <10	27 (26.5%)
>10	15 (14.7%)
Healthcare settings	
Public hospital	52 (51%)
Private	50 (49%)
Department	
Surgical	28 (27.5%)
Medical	21 (20.6%)
ER	28 (27.5%)
ICU	25 (24.5%)

### Associations between demographic characteristics and core competencies for emergency care in armed conflicts

Differences in the participants’ core competencies according to their demographic characteristics were examined using independent sample *t* tests and one‐way ANOVA (Table 2). Core competency scores did not differ significantly between male and female nurses (*p* > 0.05) or the type of the hospital (*p* > 0.05). However, the type of department significantly influenced participants’ core competencies in the context of armed conflict (*p* = 0.05). Scores were higher among those working in the medical (mean ± standard deviation: 3.93 ± 0.72) or ER (3.67 ± 0.74) departments than those working in other departments.

**TABLE 2:** The association between the demographic characteristics and core competencies for emergency care in the context of armed conflict.

Variables		Core competencies			95% CI	
	*N*	*M* (SD)	t or F	*P*	LLCI	ULCI
Gender						
Male	46	3.6 (0.68)	−0.473	0.31	−0.345	0.212
Female	56	3.66 (0.72)				
Type of hospital						
Public	52	3.58 (0.73)	−0.764	0.22	−0.384	0.170
Private	50	3.69 (0.66)				
Department						
Surgical	28	3.62 (0.64)	2.61*	0.05	3.37	3.87
Medical	21	3.93 (0.72)			3.60	4.26
ER	28	3.67 (0.74)			3.38	3.95
ICU	25	3.36 (0.63)			3.10	3.62

Based on the mean scores listed in Table 3, the top 10 competencies reflect the most valued aspects of nursing practice in disaster situations, emphasizing personal safety, patient assessment, cultural competence, recordkeeping, and immediate life‐saving interventions. These top‐ranked competencies indicate a strong focus on directly ensuring the well‐being and safety of patients and healthcare workers during emergencies. For example, nurses’ highest‐ranked competency involves the appropriate application of personal protective equipment, which is crucial for both patient and provider safety. Maintaining ongoing patient assessments and adapting care as necessary was also ranked highly, along with competencies that ensure the continuity and effectiveness of patient care, such as recordkeeping and the respectful consideration of patients’ diverse backgrounds.

**TABLE 3:** The mean differences in the core competencies based on the length of experience.

		*N*	Mean	SD	95% CI			
					Lower	Upper	*F*	*P*
Total scale	<5 years	60	3.55	0.75	3.35	3.74		
	5 to < 10	27	3.61	0.61	3.36	3.85	2.74	0.069
	>10	15	4.02	0.54	3.71	4.32		
	Total	102	3.63	0.75	3.49	3.77		
Maintain personal safety and safety of others during a disaster	<5 years	60	3.83	1.15	3.54	4.13		
	5 to < 10	27	4.00	1.03	3.59	4.41	3.12	0.048
	>10	15	4.60	0.63	4.25	4.95		
	Total	102	3.99	1.08	3.78	4.20		
Consider and respect patients’ cultures, social, and spiritual belief	<5 years	60	3.93	1.31	3.59	4.27		
	5 to < 10	27	4.26	0.85	3.92	4.60	1.10	0.336
	>10	15	4.33	1.17	3.68	4.98		
	Total	102	4.08	1.19	3.84	4.31		
Manage the post‐death care in a manner that respects the cultural, social, and spiritual beliefs of the population as each situation permits	<5 years	60	3.62	1.209	3.30	3.93		
	5 to < 10	27	3.70	1.171	3.24	4.17	0.156	0.856
	>10	15	3.80	1.265	3.10	4.50		
	Total	102	3.67	1.197	3.43	3.90		
Facilitate reverse triage regarding already admitted patients to free hospital beds, which is known as ‘surge discharging’	<5 years	60	3.48	1.282	3.15	3.81		
	5 to < 10	27	3.81	1.001	3.42	4.21	0.712	
	>10	15	3.53	1.246	2.84	4.22		0.493
	Total	102	3.58	1.206	3.34	3.82		
Maintain an ongoing assessment of patients to determine the need for a change of care	<5 years	60	3.92	1.124	3.63	4.21		
	5 to < 10	27	4.33	0.961	3.95	4.71	2.21	0.115
	>10	15	4.40	0.828	3.94	4.86		
	Total	102	4.10	1.058	3.89	4.31		
Implement appropriate nursing interventions, including emergency and trauma care, in accordance with accepted scientific principles	<5 years	60	3.67	1.271	3.34	4.00	3.11	0.049
	5 to < 10	27	4.26	0.903	3.90	4.62		
	>10	15	4.20	0.941	3.68	4.72		
	Total	102	3.90	1.165	3.67	4.13		
Understand the purpose of a disaster plan	<5 years	60	3.70	1.139	3.41	3.99	1.28	0.281
	5 to < 10	27	3.89	1.086	3.46	4.32		
	>10	15	4.20	1.014	3.64	4.76		
	Total	102	3.82	1.112	3.61	4.04		
List the appropriate steps for requesting psychological first aid for responders, patients, and other survivors	<5 years	60	3.73	1.118	3.44	4.02	1.61	0.205
	5 to < 10	27	3.70	1.068	3.28	4.13		
	>10	15	4.27	0.961	3.73	4.80		
	Total	102	3.80	1.090	3.59	4.02		
Apply the basics of infection control practices from the existing resources during disaster management	<5 years	60	3.45	1.171	3.15	3.75	1.51	0.225
	5 to < 10	27	3.89	1.013	3.49	4.29		
	>10	15	3.73	1.163	3.09	4.38		
	Total	102	3.61	1.136	3.38	3.83		
Apply critical, flexible, and creative thinking to create solutions in providing nursing care to meet the identified and anticipated patient care needs resulting from the disaster	<5 years	60	3.70	1.21	3.39	4.01	0.122	0.885
	5 to < 10	27	3.81	0.921	3.45	4.18		
	>10	15	3.67	1.05	3.09	4.25		
	Total	102	3.73	1.109	3.51	3.94		
Facilitate and perform patient transport effectively and safely during a disaster	<5 years	60	3.58	1.25	3.26	3.91	3.25	0.043
	5 to < 10	27	4.07	1.03	3.66	4.48		
	>10	15	4.33	1.11	3.72	4.95		
	Total	102	3.82	1.20	3.59	4.06		
Assist in developing recovery strategies by evaluating nursing responses during disasters to improve future disaster responses	<5 years	60	3.52	1.28	3.19	3.85	1.77	0.174
	5 to < 10	27	3.96	0.94	3.59	4.33		
	>10	15	3.93	0.88	3.44	4.42		
	Total	102	3.70	1.15	3.47	3.92		
Demonstrate the ability to understand and work within an incident management system	<5 years	60	3.58	1.27	3.25	3.91	0.052	0.949
	5 to < 10	27	3.67	1.11	3.23	4.11		
	>10	15	3.67	1.54	2.81	4.52		
	Total	102	3.62	1.27	3.37	3.87		
Demonstrate an ability to communicate with ambulance services as needed immediately	<5 years	60	3.63	1.29	3.30	3.97	2.07	0.131
	5 to < 10	27	3.78	1.42	3.21	4.34		
	>10	15	4.40	1.12	3.78	5.02		
	Total	102	3.78	1.38	3.53	4.04		
Work with appropriate individuals and agencies to assist survivors in reconnecting with their family members	<5 years	60	3.60	1.182	3.29	3.91	0.256	0.775
	5 to < 10	27	3.52	1.341	2.99	4.05		
	>10	15	3.80	1.207	3.13	4.47		
	Total	102	3.61	1.22	3.37	3.85		
Identify common human stress reactions during a disaster	<5 years	60	3.42	1.25	3.09	3.74	1.00	0.370
	5 to < 10	27	3.63	0.926	3.26	4.00		
	>10	15	3.87	1.19	3.21	4.52		
	Total	102	3.54	1.17	3.31	3.77		
Understand the components of disaster management and use them for an event, exercise, or drill	<5 years	60	3.42	1.30	3.08	3.75	0.041	0.960
	5 to < 10	27	3.33	1.18	2.87	3.80		
	>10	15	3.40	1.24	2.71	4.09		
	Total	102	3.39	1.25	3.15	3.64		
Identify vulnerable populations and to coordinate activities to reduce the risk	<5 years	60	3.50	1.03	3.23	3.77	0.037	0.963
	5 to < 10	27	3.44	1.15	2.99	3.90		
	>10	15	3.53	1.25	2.84	4.22		
	Total	102	3.49	1.08	3.28	3.70		
Recognize the disaster plan in the workplace and understand the role in the workplace at the time of a disaster	<5 years	60	3.52	1.30	3.18	3.85	2.45	0.091
	5 to < 10	27	3.67	1.03	3.26	4.08		
	>10	15	4.27	0.704	3.88	4.66		
	Total	102	3.67	1.18	3.43	3.90		
Describe the phases of the disaster management continuum: prevention/mitigation, preparedness, response and recovery/rehabilitation	<5 years	60	3.62	1.12	3.33	3.91	3.99	0.021
	5 to < 10	27	3.56	1.25	3.06	4.05		
	>10	15	4.47	0.640	4.11	4.82		
	Total	102	3.73	1.14	3.50	3.95		
Maintain and match the antidote and prophylactic medications to particular chemical or biological agents	<5 years	60	3.70	1.15	3.40	4.00	1.345	0.265
	5 to < 10	27	3.85	0.91	3.49	4.21		
	>10	15	4.20	0.94	3.68	4.72		
	Total	102	3.81	1.1	3.60	4.02		
Serve as an advocate for survivors in meeting long‐term needs	<5 years	60	3.67	1.28	3.33	4.00	0.547	0.581
	5 to < 10	27	3.41	1.31	2.89	3.93		
	>10	15	3.80	1.32	3.07	4.53		
	Total	102	3.62	1.29	3.36	3.87		
Identify and communicate important information to appropriate authorities immediately	<5 years	60	3.35	1.32	3.01	3.69	0.821	0.443
	5 to < 10	27	3.33	1.07	2.91	3.76		
	>10	15	3.80	1.37	3.04	4.56		
	Total	102	3.41	1.27	3.16	3.66		
Maintain knowledge in areas relevant to disasters and disaster nursing	<5 years	60	3.45	1.37	3.10	3.80	1.87	0.159
	5 to < 10	27	3.78	1.25	3.28	4.27		
	>10	15	4.13	1.06	3.55	4.72		
	Total	102	3.64	1.31	3.38	3.89		
Use recordkeeping processes to ensure continuity of patient information	<5 years	60	3.93	1.27	3.60	4.26	1.38	0.255
	5 to < 10	27	3.93	1.07	3.50	4.35		
	>10	15	4.47	0.64	4.11	4.82		
	Total	102	4.01	1.15	3.78	4.24		
Use specific communication tools during a disaster	<5 years	58	3.43	1.23	3.11	3.75	1.50	0.228
	5 to < 10	26	3.35	1.26	2.84	3.86		
	>10	15	4.00	1.25	3.31	4.69		
	Total	99	3.49	1.25	3.25	3.74		
Identify human behaviors that put individuals at risk during a disaster	<5 years	60	3.60	1.14	3.31	3.89	3.41	0.037
	5 to < 10	27	3.52	.975	3.13	3.90		
	>10	15	4.33	0.724	3.93	4.73		
	Total	102	3.69	1.07	3.48	3.90		
Determine the need for decontamination, isolation, or quarantine and take appropriate actions	<5 years	60	3.47	1.42	3.10	3.83	0.691	0.504
	5 to < 10	27	3.41	1.08	2.98	3.84		
	>10	15	3.87	1.06	3.28	4.45		
	Total	102	3.51	1.28	3.26	3.76		
Understand terminology relevant to disasters	<5 years	60	3.53	1.36	3.18	3.88	1.79	0.172
	5 to < 10	27	3.44	0.934	3.08	3.81		
	>10	15	4.13	0.915	3.63	4.64		
	Total	102	3.60	1.213	3.36	3.84		
Understand the nursing laws, policies, and procedures relevant during a disaster	<5 years	60	3.78	1.136	3.49	4.08		
	5 to < 10	27	3.70	1.137	3.25	4.15	1.40	0.251
	>10	15	4.27	0.884	3.78	4.76		
	Total	102	3.83	1.11	3.62	4.05		
Participate in creating new guidelines for nursing practice in disaster	<5 years	60	3.80	1.02	3.54	4.06	2.963	0.056
	5 to < 10	27	3.59	1.21	3.11	4.07		
	>10	15	4.40	0.737	3.99	4.81		
	Total	102	3.83	1.06	3.62	4.04		
Identify the signs and symptoms of exposure to CBRNe agents	<5 years	60	3.47	1.37	3.11	3.82	0.911	0.406
	5 to < 10	27	3.11	1.37	2.57	3.65		
	>10	15	3.67	1.6	2.79	4.55		
	Total	102	3.40	1.4	3.13	3.68		
Participate in processes of securing adequate personnel, supplies, equipment, and space for patient care, which is also known as “surge capacity”	<5 years	60	3.77	1.14	3.47	4.06	3.73	0.027
	5 to < 10	27	3.30	1.07	2.87	3.72		
	>10	15	4.27	1.16	3.62	4.91		
	Total	102	3.72	1.16	3.49	3.94		
Describe strategies for allocating scarce resources in an ethical manner to optimize population outcomes during triage and treatment	<5 years	60	3.13	1.26	2.81	3.46	2.15	0.122
	5 to < 10	27	3.11	0.89	2.76	3.46		
	>10	15	3.80	1.2	3.13	4.47		
	Total	102	3.23	1.18	2.99	3.46		
Understand the major classes of CBRNe agents	<5 years	60	2.98	1.16	2.68	3.28	2.33	0.102
	5 to < 10	27	2.81	1.11	2.38	3.25		
	>10	15	3.60	1.24	2.91	4.29		
	Total	102	3.03	1.17	2.80	3.26		
Describe the principles of crisis communication in crisis intervention and risk management	<5 years	60	3.32	1.13	3.03	3.61		
	5 to < 10	27	3.15	1.1	2.71	3.58	1.27	0.283
	>10	15	3.73	1.3	3.02	4.44		
	Total	102	3.33	1.14	3.11	3.56		
Understand and adhere to the plan in case of hospital functional collapse due to a shortage of fuel or electricity shutdown	<5 years	60	3.27	1.31	2.93	3.61	1.57	0.212
	5 to < 10	27	2.96	1.02	2.56	3.37		
	>10	15	3.67	1.29	2.95	4.38		
	Total	102	3.25	1.25	3.00	3.49		
Provide up‐to‐date information to the disaster response team regarding healthcare issues and resource needs	<5 years	60	3.25	1.1	2.96	3.54	3.51	0.034
	5 to < 10	27	3.26	1.0	2.85	3.66		
	>10	15	4.07	1.16	3.42	4.71		
	Total	102	3.37	1.13	3.15	3.59		
Provide defensible solutions to a series of ethical dilemmas arising in a disaster	<5 years	60	3.50	1.2	3.19	3.81	1.34	0.264
	5 to < 10	27	3.07	0.92	2.71	3.44		
	>10	15	3.47	1.2	2.81	4.12		
	Total	102	3.38	1.14	3.16	3.61		
Manage and supervise volunteers	<5 years	60	3.35	1.35	3.00	3.70	2.82	0.064
	5 to < 10	27	3.63	1.12	3.19	4.07		
	>10	15	4.20	1.08	3.60	4.80		
	Total	102	3.55	1.3	3.30	3.80		
Work in different geographical areas with unknown or foreign colleagues	<5 years	60	3.53	1.33	3.19	3.88	1.86	0.160
	5 to < 10	27	3.70	1.41	3.15	4.26		
	>10	15	4.27	1.0	3.69	4.84		
	Total	102	3.69	1.33	3.43	3.95		
Develop and maintain a personal and family preparedness plan	<5 years	60	3.58	1.41	3.22	3.95	2.26	0.109
	5 to < 10	27	3.93	0.87	3.58	4.27		
	>10	15	4.33	1.4	3.56	5.11		
	Total	102	3.78	1.3	3.53	4.04		
Use appropriate personal protective equipment properly	<5 years	60	4.18	0.813	3.98	4.39	0.572	0.566
	5 to < 10	27	4.14	0.601	3.91	4.38		
	>10	15	3.93	1.1	3.32	4.54		
	Total	102	4.13	0.81	3.98	4.29		
Provide the principles of first aid immediately as needed during a disaster	<5 years	60	3.61	0.83	3.40	3.82	0.698	0.500
	5 to < 10	27	3.40	0.75	3.11	3.70		
	>10	15	3.46	0.83	3.0	3.92		
	Total	102	3.53	0.80	3.38	3.69		
Prioritize patients to maximize survival	<5 years	60	3.56	0.87	3.34	3.79	0.318	0.729
	5 to < 10	27	3.62	0.93	3.26	3.99		
	>10	15	3.40	0.97	2.85	3.94		
	Total	102	3.55	0.89	3.38	3.73		
Manage burns, blast, and crush injuries during a disaster	<5 years	60	3.10	0.93	2.85	3.34	1.45	0.240
	5 to < 10	27	3.03	1.05	2.61	3.45		
	>10	15	3.53	0.91	3.02	4.04		
	Total	102	3.14	0.97	2.95	3.33		
Perform decontamination following appropriate procedures	<5 years	60	2.88	0.74	2.69	3.07	0.539	0.585
	5 to < 10	27	3.07	0.96	2.69	3.45		
	>10	15	3.00	0.85	2.53	3.46		
	Total	102	2.95	0.813	2.79	3.11		

The lower‐ranked items may reflect competencies that, while still important, may not be at the forefront of the emergency response or are not directly related to immediate life‐saving procedures. For example, tasks such as identifying behaviors that put individuals at risk during a disaster, cultural sensitivity when managing post‐death care, and understanding the roles within a disaster plan are perceived as less immediate or situational within the context of disaster nursing.

Differences in core competency scores according to length of experience were examined using one‐way ANOVA (Table 3). More experienced nurses (> 5 years of experience) believed more about implementing appropriate nursing interventions by accepted scientific principles than newly recruited nurses (< 5 years of experience). In addition, compared with nurses with < 10 years of experience, nurses with ≥10 years of experience had greater perceptions toward maintaining patient safety during a disaster (*p* < 0.05), facilitating and performing patient transport effectively and safely during a disaster (*p* < 0.05), describing the phases of disaster management (*p* = 0.021), identifying human behaviors that put individuals at risk during a disaster (*p* = 0.03), creating “surge capacity” (*p* = 0.02), and providing up‐to‐date information to the disaster response team (*p* = 0.03).

## DISCUSSION

The ranked nursing core competencies in armed conflict situations revealed a dual focus on immediate, life‐saving actions, and maintaining personal safety. The high ranking of competencies associated with personal protective equipment, patient assessment, and cultural sensitivity underscores a prevailing emphasis on direct patient care during emergencies, which is consistent with other studies (Mani et al., 2023a, 2023b). This observation reflects the nurses’ acute awareness of the critical “golden moments” in which immediate interventions are necessary to save lives and ensure the safety of healthcare providers. However, the low ranking of some competencies suggests a potential underestimation of the importance of broader disaster management skills. Although crucial, competencies such as understanding workplace disaster plans, culturally sensitive post‐death care, and risk behavior identification are perceived as less immediate. This perception might indicate nurses’ intense focus on the clinical aspects of emergency response to the detriment of other vital functions in disaster situations.

Our study highlights that the intense immediacy of disaster response often deprioritizes strategic thinking and long‐term planning. This critical competency gap can lead to a fragmented disaster response, potentially undermining the effectiveness of long‐term recovery and rehabilitation efforts. Notably, competencies associated with managing post‐death care, identifying risk behaviors, and understanding disaster roles are integral to a holistic approach to disaster management, which extends beyond the response phase to include preparedness, mitigation, and recovery (Bou‐Karroum et al., 2020; Levy & Sidel, 2016; Mani et al., 2023b). Our findings highlight a need for disaster management training to ensure that nurses are well‐rounded in their approach to managing disasters. There is an opportunity for institutions to incorporate relevant instructional methods, including augmented reality and other advanced technologies, into training programs as a method to enhance competencies across the disaster management continuum (Chen & Liou, 2023).

Our results also showed departmental differences in competency scores, suggesting that the context nurses operate in may shape their perspectives on disaster competencies. Nurses from the medical and ER departments rated some core competencies higher than their counterparts, potentially reflecting more frequent engagement with emergencies. Because different departments value different skills, training programs must be customized. Each program should focus on the specific skills needed in a particular department or healthcare setting.

Although cultural and ethical competencies are recognized as critical in disaster nursing (Al Thobaity et al., 2016; International Council of Nurses, 2019; Mani et al., 2023b), they were ranked comparatively lower in perceived importance by our study participants. This finding highlights a potential disconnect between recognized best practices and the perceived priorities of nurses in this specific context. Future research should explore targeted training interventions to strengthen these competencies and investigate the impact of such interventions on the quality of care delivered during disaster situations. Understanding this relationship is essential for developing comprehensive and culturally sensitive disaster preparedness programs.

Our findings highlighted a strong association between years of nursing experience and perceived competency in disaster‐related care. More experienced nurses (> 10 years of experience) consistently demonstrated greater confidence across essential disaster nursing competencies than less experienced nurses. This observation is particularly evident in areas such as maintaining patient safety during disasters, where experienced nurses (4.60 ± 0.63) significantly outscored less experienced nurses (*p* < 0.05). This finding is consistent with the understanding that extensive clinical experience cultivates a heightened awareness of potential risks and strengthens the ability to anticipate and mitigate threats to patient well‐being in high‐pressure situations (Mani et al., 2023a).

Furthermore, our study revealed that nurses with ≥5 years of experience believed more in their ability to implement evidence‐based interventions than newly recruited nurses (< 5 years of experience). This difference likely reflects the accumulated knowledge and practical application of best practices that nurses acquire over time. The importance of experience extends beyond direct patient care. Nurses with > 10 years of experience also reported greater confidence in managing logistical aspects of the disaster response, including patient transport, disaster management phases, identifying risk‐prone behaviors, and establishing surge capacity (all *p* < 0.05). This observation suggests that prolonged exposure to diverse clinical scenarios and potential disaster drills contributes to a more comprehensive understanding of disaster management principles and their practical application.

### Implications for nursing and health policy

Our study has significant implications for both nursing practice and health policy, particularly in conflict‐affected regions. Our findings underscore the need for targeted training programs that address nurses’ self‐identified competency gaps in managing complex trauma, psychological trauma, and resource‐limited settings. Integrating simulation‐based learning, mentorship opportunities, and mental health support into the nursing curriculum and continuing education is crucial. Furthermore, health policies should prioritize resource allocation toward improving emergency care infrastructure, equipment, and staffing levels in conflict zones.

A multifaceted approach is crucial to building a resilient and responsive nursing workforce in challenging environments like Yemen, including implementing competency‐based deployment strategies to ensure that nurses are placed in roles that match their skills and experience, maximizing their effectiveness and job satisfaction. Equally important is the provision of robust mental health services to support nurses in coping with the stress and emotional toll often associated with working in demanding and resource‐constrained settings. Finally, ongoing data collection and analysis are essential to inform evidence‐based policymaking, ensuring that healthcare workforce initiatives are tailored to the specific needs and challenges of the context.

### Limitations

Although this study is the first of its kind in such an underdeveloped country as Yemen, it has some limitations. First, there are limitations inherent to its cross‐sectional design and sample size, which may limit the generalizability of its findings. Second, although it used this competency questionnaire, it did not formally evaluate its psychometric properties, potentially impacting the accuracy of competency measurements. However, this adopted competency questionnaire was developed from a previously validated instrument that underwent rigorous psychometric testing by Al Thobaity et al. (2016), who demonstrated its reliability and validity, supporting the use of the adopted questionnaire in our study. As outlined in Mani et al. (2023b), only minor adaptations were made to tailor this questionnaire to the specific demands of armed conflict, preserving its core elements and measurement properties.

## CONCLUSIONS

Our findings highlight a significant issue in nursing preparedness for armed conflict situations in Yemen. Despite the high value placed on clinical skills for immediate patient care, a noticeable deficiency exists in training for wider mass casualties’ management responsibilities. The identified disparities suggest that while nurses feel confident in their ability to provide direct care, they may be less prepared for the strategic and operational aspects of disaster response, such as triage management, logistics, and long‐term patient care planning. Moreover, the length of nursing experience emerged as a crucial factor influencing perceived competency in disaster nursing. This finding emphasizes the need for training programs to consider nurses’ experience and tailor educational interventions to address the specific needs of less experienced nurses, potentially through mentorship programs or targeted simulations, to ensure a workforce uniformly prepared for disaster response.

## AUTHOR CONTRIBUTIONS

*Study conception/design*: Zakaria Ahmed Mani, Adnan Innab, and Fuad Taleb. Data collection: Fuad Taleb. *Data analysis*: Zakaria Ahmed Mani, Adnan Innab, and Fuad Taleb. *Study supervision*: Zakaria Ahmed Mani and Adnan Innab. *Manuscript writing*: Zakaria Ahmed Mani, Adnan Innab, and Fuad Taleb. *Critical revisions for important intellectual content*: Zakaria Ahmed Mani, Adnan Innab, and Fuad Taleb.

## CONFLICT OF INTEREST STATEMENT

The authors declare no conflicts of interest.

## DISCLAIMER

The authors declare that they have no competing financial interests or personal relationships that may have influenced the work reported in this study.

## Data Availability

The data supporting the findings of this study are available from the corresponding author (Zakaria Ahmed Mani) upon reasonable request.
